# Shame, health literacy and consent

**DOI:** 10.1177/14777509231218203

**Published:** 2023-12-12

**Authors:** Barry Lyons, Luna Dolezal

**Affiliations:** 1 School of Medicine, Trinity College Dublin, Dublin, Ireland; 2 Anaesthesia & Critical Care, Children’s Health Ireland, Dublin, Ireland; 3 Wellcome Centre for Cultures and Environments of Health, University of Exeter, Exeter, UK

**Keywords:** Informed consent, patient relationships, health literacy, shame

## Abstract

This paper is particularly concerned with shame, sometimes considered the ‘master emotion’, and its possible role in affecting the consent process, specifically where that shame relates to the issue of diminished health literacy. We suggest that the absence of exploration of affective issues in general during the consent process is problematic, as emotions commonly impact upon our decision-making process. Experiencing shame in the healthcare environment can have a significant influence on choices related to health and healthcare, and may lead to discussions of possibilities and alternatives being closed off. In the case of impaired health literacy we suggest that it obstructs the narrowing of the epistemic gap between clinician and patient normally achieved through communication and information provision. Health literacy shame prevents acknowledgement of this barrier. The consequence is that it may render consent less effective than it otherwise might have been in protecting the person’s autonomy. We propose that the absence of consideration of health literacy shame during the consent process diminishes the possibility of the patient exerting full control over their choices, and thus bodily integrity.

## Introduction

While emotions are common in healthcare environments, where experiences such as worry, fear, grief, hope and anxiety are self-evidently commonplace, there has been a cultural tendency in clinical medicine to regard patients’ affective experiences as irrelevant or superfluous to the task at hand – namely curing biological disease or malfunction. As Sternberg observes, ‘the notion that emotions could have something to do with disease came to be viewed by modern science with disdain and mild amusement’.^1^ However, understanding the ‘affective climate’, or the particular constellation of emotions that are commonly produced and experienced in a healthcare environment, has been demonstrated to be key to not only an understanding of the first-person experience of patients and to the decisions that they make, but is also pertinent to outcomes.^2^

Informed consent is one area of healthcare practice where the relevance of emotions to healthcare decision-making has been neglected, the consequence of a particular emphasis on rationality and agency. In general, the legal and regulatory provisions around consent are formulated with a focus on procedural elements and patient capacity. For reasons of self-protection in the event of complaint or litigation, clinicians largely feel compelled to adhere to this legal construction, although they might fall short of it in practice. Drobac and Goodenough argue that the rigid legal model of consent runs ‘in tension with reality’^3^; that although our prevailing legal conceptions are useful for ‘ease of legal administration and social organization’, the everyday actuality of doctor-patient interactions is replete with flawed information, inexperience, inattentiveness, and cognitive imperfection rendering the real world of consent as being somewhat different. To the list of Drobac and Goodenough, we would add the emotional context within which the consent discussion takes place.

Amongst others, Supady *et al*. propose a significant relationship between the affective and cognitive components of emotions, and the processing of information and decision-making required of the patient during the informed consent process.^4^ Although the law does not appear to deliberately exclude consideration of psychological or emotional matters in the consent transaction between doctor and patient, these are not factors that tend to be mentioned in guidance around the requirements or process of consent. We suggest that the absence of exploration of affective issues during the consent process is problematic. Emotions commonly impact upon our decision-making process and some, such as fear, anger and disgust, have been shown to have a significant influence on choices related to health and healthcare.^5^ This paper is particularly concerned with shame, sometimes considered the ‘master emotion’,^6^ and its possible role in affecting the consent process, specifically where that shame relates to the issue of diminished health literacy.

Patients who have reduced health literacy may have difficulties employing the cognitive or social skills that are traditionally relied upon to access, navigate, comprehend and use information that is important for the maintainance and promotion of their health status.^7^ While they may well recognise their disadvantage in respect of interpreting or understanding presented medical information, they can feel precluded from revealing their difficulty because they suffer from a sense of shame at their own perceived inadequacy. In one study, 40% of patients who acknowledged their own low health literacy admitted to feeling ashamed by, and to hiding, their inability to read.^8^

Despite the healthcare environment being one where patient shame is easily incited or exacerbated, shame remains an ‘elephant in the room’, rarely acknowledged, or addressed as relevant to outcomes.^9^ Although there is little or no evaluation of how shame might affect or interfere with processes of informed consent, we propose that it can have a significant impact, primarily mediated through self-presentation and discourse with healthcare professionals. We are not arguing that shame renders an individual incapable of making decisions; rather we are making the more modest claim that experiencing shame in the healthcare environment may close off discussions and alternatives. In the case of impaired health literacy we suggest that it obstructs the narrowing of the epistemic gap between clinician and patient normally achieved through communication and information provision. Health literacy shame prevents acknowledgement of this barrier. The consequence of shame in this instance is that it may render consent less effective than it otherwise might have been in protecting the person’s autonomy. And thus such individuals are at risk of having their values compromised, or even of making decisions harmful to themselves. We would propose that the absence of consideration of shame, and other emotions, during the consent process diminishes the possibility of the patient exerting full control over their choices, and thus over their bodily integrity.

Although we predominantly refer to the law of England and Wales in this paper this is largely for illustrative purposes. While there are some inter-jurisdictional differences in respect of legal or professional rules around medical consent, such variations are not relevant to the issues central to this article. The subject of impaired health literacy and its relationship to medical consent should be, we would suggest, a concern for many countries.

## Consent

The generally accepted rules around consent to any healthcare intervention entail that it must be voluntary, informed and provided by someone with decision-making capacity. A deficiency in any one of these three elements may vitiate consent. Asking for permission before infringing on another’s body meets not just legal and regulatory requirements, but is also regarded as morally transformative in the sense that consent vests the control over bodily integrity in the patient, thus respecting their autonomy.^10^

The importance of the second of these elements, communication and information-giving, was affirmed by the UK Supreme Court in *Montgomery v Lanarkshire Health Board*.^11^ The basic information that needs to be disclosed to the patient for consent to be valid relates to the benefits of the proposed intervention and the risks attached, as well as any reasonable alternative options. Obviously, the demands that this places on both practitioner, in information giving, and patient, as interpreter of the information received, escalate as the complexity and seriousness of a procedure increases. The completeness of information provision is an attempt to minimise epistemic inequality between practitioner and patient. Effective discussions are likely to reveal patient preferences and ensure that the ultimate care plan aligns with the patient’s values.

A second issue central to the validity of consent is the notion of mental capacity, of the competence of the individual patient to make a particular decision in respect of some healthcare intervention.^12^ In general, patients are presumed to have the capacity to act as decision-makers in respect of their own healthcare needs. Where there is doubt, a standard legal test is applied to assess capacity. As set out in the Code of Practice attached to the Mental Capacity Act 2005,^13^ the test involves two stages, the first assessing for the presence of an impairment of, or a disturbance in the functioning of, their mind or brain. If affirmative, the second asks whether the impairment or disturbance affects the person in such a way that they are thus unable to make a specific decision when they need to. Where the question arises, a person is deemed competent to make a decision if they can understand information about the decision to be made; retain, use and weigh that information as part of the decision-making; and communicate their decision.

While the criteria of information and capacity obviously have validity in the sense that they are the applied, and expected, standards within a medico-legal framework, they offer an incomplete picture of human decision-making. In particular, the process described is exclusively cognitive and focused on procedural matters: was the patient competent to consent; did the practitioner provide the relevant information; was the transaction effectively documented? In essence, the law portrays the standard patient in the consent process as *homo economicus* – an emotionless, calculating machine that is perfectly rational and self-interested. Such individuals are straightforwardly capable of internalising substantial amounts of complex information, of weighing the costs and benefits of each possible choice, and of making logical and wise decisions based on probability even under conditions of uncertainty.^14^

This model is substantially derived from a Millian liberal view of autonomy, of the individual operating within a sphere of freedom protected from state interference. Arguing from the perspective of mental health law, Donnelly contends that this view of individual agency is flawed in that it neglects to take account of the real-world context within which people take decisions. The model primarily values the right to be left alone, and thus fails people who are vulnerable because of social, cultural or economic disadvantage as the presumption of unfettered autonomous decision-making may not represent their reality.^15^

It seems reasonable to hold that even individuals without any of these listed disadvantages may fall short of the Millian ideal of agency through the turmoil wrought from the stress and emotional upheaval induced by serious illness–what Virgina Woolf described as the uprooting of ‘ancient and obdurate oaks’ within us.^16^ The law frequently seems oblivious to this, or at least is silent on the subject. Donnelly also borrows from Virginia Woolf to illuminate this point. The law, she states^17^:

does its best to maintain that its concern is with the mind; that the body is a sheet of plain glass through which the soul looks straight and clear, and, save for one or two passions … is null, negligible and non-existent. On the contrary, the very opposite is true.^18^

Akin to Donnelly, there seems reasonably common ground across many humanities disciplines that emotions routinely impact upon the choices we make in general, and upon healthcare decision-making in particular. As an example, a sense of overwhelming love may lead a parent to commit to being a live organ donor for their sick child in desperate need of a kidney transplant without any proper or rational analysis of the risks involved. Indeed, in breach of the orthodox decisional arc of *‘information–reasoning–choice’*, early work by Fellner and Marshall indicates that donors engaged in a decision-making process that was immediate, irrational, and contrary to the normal requirements for informed consent: ‘not one of the donors weighed alternatives and rationally decided’.^19^ The decisions in this instance appear likely to have been made in a peremptory way on the basis of familial obligations and emotional responses.

This is entirely in keeping with a standard understanding of how people make choices – we elect to make one decision as opposed to another on the basis of different sorts of values that influence the development of our goals and preferences. Emotions are a recognisable and important source of such values, especially when it comes to medical decisions.^20^ Simmerling contends that emotions should be considered an essential ingredient of competence, citing Charland who argues that ‘emotions form a class of recognizable reasons of their own and that competence to consent … is a matter of practical, rather than theoretical reasoning’.^21^ Commenting on Fellner and Marshall’s observations, Simmerling suggests that the problem with emotions lies in the perception that affective responses indicate a lack of competence, that the conception of agency equates freedom of choice with an independence from emotional or moral commitments.^22^

Occasionally the courts have recognised that matters related to the patient’s state can have an impact on consent. In *Re T* (*Adult: Refusal of Medical Treatment*) Lord Donaldson specified that a person’s capacity may be reduced ‘by reason of temporary factors, such as unconsciousness or confusion or other effects of shock, severe fatigue, pain or drugs being used in their treatment’,^23^ these findings being reinforced in a small number of subsequent cases.^24^ The courts do seek to downplay the importance of these matters, finding that only where these ‘completely erode capacity’ should they be found that consent is vitiated.^25^

For law and medicine, the consequences of acknowledging the impact of emotions on healthcare decision-making may be the disruption of the legal fiction of consent alluded to by Drobac, and others.^26^ Thus, while it is notable that the above legal judgements fall short of intensely evaluating or interrogating the role of particular affective issues on decision-making, this is unsurprising. To an extent we are in agreement with this position – we are not proposing that the presence of strong emotions should be interpreted as indicating that the patient lacks the capacity to consent. Nonetheless, it should be recognised that emotions such as shame, can significantly affect the decision-making process, impacting upon how information provided can land and be interpreted, on what is retained, what is lost, and what questions are asked.

## Phenomenology of shame in healthcare

Shame is a self-conscious, cognitive affective construct; an emotion that arises in response to an evaluation of the self. It is characterised by negative judgements, where the self is appraised as inherently flawed, inadequate, undesirable or simply bad.^27^ Shame and its cognates, embarrassment and humiliation, are very common affective accompaniments to experiences like illness, nudity or disfigurement. As set out in Aaron Lazare’s seminal article on the subject:

patients may experience physical or psychologic limitations as defects, inadequacies, or shortcomings … Treatments and their side effects may be potential sources of further shame and humiliation: mastectomies, the loss of hair, and impotence are examples.^28^

Experiences like this, where a patient feels vulnerable, flawed, exposed or judged, are prevalent in healthcare and may lead to a variety of health-relevant behaviours or actions.^29^ Shame plays an important role in how patients interact with health care providers, and is a powerful driver of decision-making that affects health-relevant behaviour, thus having concrete effects on clinical out-comes.^30^ Even just the anticipation of shame can provoke a range of avoidance or protective behaviours that can impact negatively on health-seeking behaviour, or engagements with healthcare professionals. Responses to the threat of shame, where an individual is trying to protect themselves or their reputation or status, commonly involve withdrawal, hiding or avoidance.^31^

What Lazare points out is that body shame is routine in medicine, where patients may be self-conscious, embarrassed or ashamed about the physical body and its ‘failings’, or deviations from a norm of health in terms of appearance or function. Beyond the inherent possibility for patients to feel shame about their bodies or physical conditions, clinical encounters themselves are inherently shame-producing. As Salter and Hall argue, professional practices like medicine and social care are frequently ‘vectors of shame, humiliation, and inequality’.^32^ They are situations of vulnerability, settings where feelings of self-consciousness or shame are readily incited and easily exacerbated. Interactions with healthcare professionals often involve unequal power relations, and exposure, scrutiny and judgment of an individual’s body, lifestyle and circumstances, along with other vulnerabilities related to their mental or physical health.^33^

Shame, as Bernard Wiliams points out, produces ‘necessity’, a deeply rooted need for avoidance of situations or circumstances where there is the potential to experience the emotion, or shameful exposure.^34^ It can, he suggests, feel like a matter of life or death to avoid locations, events or interactions that hold this possibility. There is evidence that this ‘necessity’ can interfere with individuals accessing healthcare and negatively affect the quality of care they ultimately receive.^35^ In the context of seeking or needing healthcare, individuals who are anxious about shameful exposure may therefore avoid seeking help in the first place, may miss appointments, may sidestep disclosing honest details about symptoms, lifestyle or circumstances, may fail to follow through with treatments and may conceal diagnoses and coping behaviours from friends, family and professionals. And because the identification of shame is itself shameful, and can lead to feelings of further embarrassment or humilitiation when acknowledged, addressing shame directly is also routinely avoided in clinical and therapeutic encounters.^36^ Thus, shame and related emotions frequently remain under-emphasized or entirely unappreciated, with clinicians still not trained to understand shame or its effects.^37^

While the most obvious instances of health-related shame arise from the body or illness, non-corporeal shame may also arise in healthcare contexts, and hence be health-relevant. For instance, shame related to poverty, literacy levels, engagement with the criminal justice system, displacement, immigration status, trauma, class, other social dynamics or personal circumstances, may be a significant factor in how an individual interacts with healthcare professionals and services.^38^

## Shame and health literacy

Decreased health literacy is one situation where shame, and the ‘necessity’ for shame avoidance, may be particularly problematic. Notwithstanding our critique of the procedural essence of the consent process, the provision of sufficient information about the proposed procedure is of immense importance. The purpose of this exchange is to reduce the power imbalance between clinician and patient, and enhance the patient’s understanding and, hence, control over the situation. However, for this to be given maximal effect, the information, either verbal or written, must be provided in a manner that is comprehensible to the patient. For patients with diminished mental capacity, legislation in some jurisdictions insists that doctors go to significant efforts to ensure the individual understands the given information.^39^ Despite the need seeming obvious, it is unlikely that patients without ‘a disease of the mind or brain’ will be afforded the same consideration. In general, there is an assumption that patients who are ‘normal’ are capable of understanding, internalising and reasoning with standardly provided medical information.

However, this presumption is problematic. The relevance of achievement in reading, writing and communication skills is not simply that it has educational value, rather what is more important is what such proficiency enables us to do.^40^ Health literacy is defined by the World Health Organization as ‘the cognitive and social skills which determine the motivation and ability of individuals to gain access to, understand and use information in ways which promote and maintain good health’.^8^ It entails the basic capacities required to obtain, process and understand basic health information, and to navigate the health system and its component services, in order to make appropriate health decisions.^41^

A standard categorisation of literacy competence has been set out as follows^41^:

1. Basic/functional literacy
2. Communicative/interactive literacy
3. Critical literacy

The first, functional literacy, relates to the basic skills of reading, writing and communication that allow for efficient functioning in everyday situations. The second, critical literacy, involves the capacity for effective analysis and use of information in order to be in control of life events in so far as is possible. Finally, interactive literacy permits the extraction of information and the derivation of meaning from different forms of communication, and the application of new information to changing circumstances. A wide range of health tasks and interactions depend on health literacy, including those related to health promotion, health protection, disease prevention, personal health care and health maintenance, and health system navigation.^42^

Without going into detail, this classification indicates that the different levels of health literacy progressively allow for the exercise of greater autonomy. Advancing between the categories improves people’s capability to access health information and their capacity to use it effectively. Progression to the highest level attainable is critical to personal empowerment.^43^ Conversely, lack of adequate literacy represents an important barrier to receiving high quality care. According to the Institute of Medicine, individuals with literacy difficulties are likely to have substantial problems obtaining, processing and understanding the basic information and services needed to make appropriate health decisions.^44^

There is substantial evidence that limited literacy is a significant risk factor for poor health outcomes. Across two temporally separate systematic reviews, Berkman *et al*. concluded that low health literacy was associated with poorer health-related knowledge and comprehension,^45^ with the second review identidying that it is also associated with differential use of certain health care services, including decreased mammography screening and influenza immunization utilisation, but increased accessing of emergency care and hospitalisation.^42^ There are also the obvious difficulties in interpreting medication labels, and a consequent reduction in taking medications properly (e.g., right dose at the right time for the right duration). All of this leads to poorer overall health status and health-related outcomes, and a higher mortality rate, particularly amongst elderly persons.

Unfortunately, diminished health literacy is a common problem.^9^ Amongst a predominately indigent African-American community in the United States, Parikh *et al*. evaluated that over 40% had inadequate or marginal functional health literacy, with a correlation between lower literacy and male gender, older age and lower educational attainment. The European Health Literacy Survey found that almost one in two citizens (47%) had limited (insufficient or problematic) health literacy,^46^ although the level differed substantially between countries, from 29% in the Netherlands to 62% in Romania. Akin to Pariks’s work, European literacy levels were aligned with a social gradient – lower levels being related to those subgroups within a population who are ‘defined by financial deprivation, low social status, low education or old age’.

However, low health literacy is frequently undisclosed – many patients in Parikh’s study had never divulged their illiteracy even to their spouse or other family members. Many of these admitted to feeling shame in response to their perceived inadequacy and therefore kept it hidden.^9^ Health literacy shame may also be additive, an augmentation of the stigma and shame already experienced as a consequence of poverty.^47^ Because of shame, people with impaired health literacy may not bring anyone (who might assist them) with them to medical appointments. Shame impedes their asking for help in keeping with the well documented phenomenological experiences of hiding, withdrawal and avoidance. Even the matter that they have hidden their literacy difficulties and ‘pretended’ to their families in order to ‘pass’ is potentially shameful.

Thus, in striving to avoid the possibility of shameful exposure, patients with lower literacy are more likely to pretend that they understand the documentation and discussions relevant to their health. There is a cost to this as they potentially jeopardise their own treatment, and well-being. This might mean signing forms that they have not been able to read, or consenting to procedures without understanding sufficient information to make a balanced and personal decision in order to avoid questioning, scrutiny or risk of exposure. Some patients may even avoid forms of care altogether because of embarrassment about their illiteracy.^48^

## Unintended consequences: shame and literacy tests

In response to this there has been a debate over whether literacy tests should be used routinely in clinical practice to screen patients for potential problems, and also the suggestion that a patient’s reading ability should be recorded on their medical records.^49^ The benefits of literacy screening, and flagging lower literacy to healthcare providers, seem apparent.^50^ However, identifying patients as having limited literacy may result in unintended consequences, such as a feeling of stigmatisation and the consequent potential avoidance of health services. Empirical data indicates that a significant number of patients have reported they would feel ashamed if medical staff knew of their limited literacy.^49^ In this instance, shame compounds the barriers to health already wrought by poverty, social disadvantage, and of course, low health literacy.

None of this should be surprising. Literacy testing and acknowledgement of impaired literacy seems on the surface to be a straightforward good if it results in the tailoring of how information is provided to meet the needs of the recipient. The cost of shame is that the ‘necessity’ to avoid exposure renders this problematic as patients may seek to evade testing, or any admission of impaired literacy, even though this could go against their own best interests, and involves potentially disadvantaging or harming themselves.

## Conclusion

The potential negative impact of low health literacy on patients in relation to informed consent seems obvious, although specific empirical data is lacking. Our concern is that some patients, who may already be disadvantaged within the social and health care setting, will come to make health choices that are different to the decisions they would have made if they had full awareness and understanding of all of the possibilities. Testing for reduced health literacy may seem a practical solution, but it is easy to see how it could further stigmatise and shame an already vulnerable population.

One frequently articulated solution is that organisational health service settings develop health literacy policies that promote engagements, education processes and service delivery strategies that are appropriate for people with different health literacy needs. Such approaches would avoid the need for a health service to evaluate the health literacy of every patient, but rather ensure that clinicians understand the issues surrounding health literacy, and possess the range of relevant skills to engage in good practice related to it.^51^ Prominent advocates of this approach argue for the need to ‘structure the delivery of care as if every patient may have limited health literacy’.^52^

Much of the data to date provides an understanding of how reduced health literacy affects patients clinically, but not emotionally. Thus, while we agree with the above proposal, we suggest that alone it is insufficient. Rather it is but one part of what might overall be considered as a need for enhanced cultural competence in medicine.^53^ We see cultural competence as not just being related to matters such as ethnic or religious differences, social disadvantage or migrant status (important as these undoubtedly are), but also seeks to identify and understand the role of emotion in the patient experience. This requires the adaptation of a phenomenological approach that aims to narrow the gap between objective care delivery and subjective experiences which are multiple and varied.^54^ Incorporating the teaching of phenomenologically-derived emotionally sensitive practice into the medical education curriculum is ultimately likely to lead to the development of a more attuned dialogue between physicians and patients. In the domain of consent, the acknowledgement that emotion affects decision-making offers the possibility of devising interventions that improve personal choice, and vindicate patient rights.

## Data Availability

Data sharing not applicable to this article as no datasets were generated or analyzed during the current study.
